# Small Intestinal Bacterial Overgrowth in Children: A State-Of-The-Art Review

**DOI:** 10.3389/fped.2019.00363

**Published:** 2019-09-04

**Authors:** David Avelar Rodriguez, Paul MacDaragh Ryan, Erick Manuel Toro Monjaraz, Jaime Alfonso Ramirez Mayans, Eamonn Martin Quigley

**Affiliations:** ^1^Pediatric Gastroenterology and Nutrition Unit, National Institute of Pediatrics, Mexico City, Mexico; ^2^School of Medicine, University College Cork, Cork, Ireland; ^3^Lynda K. and David M. Underwood Center for Digestive Disorders, Houston Methodist Hospital, Houston, TX, United States

**Keywords:** small intestine bacterial overgrowth, small intestinal bacterial overgrowth, small bowel bacterial overgrowth, proton pump inhibitors, gut microbiota, stunting

## Abstract

Small intestinal bacterial overgrowth (SIBO) is a heterogenous and poorly understood entity characterised by an excessive growth of select microorganisms within the small intestine. This excessive bacterial biomass, in turn, disrupts host physiology in a myriad of ways, leading to gastrointestinal and non-gastrointestinal symptoms and complications. SIBO is a common cause of non-specific gastrointestinal symptoms in children, such as chronic abdominal pain, abdominal distention, diarrhoea, and flatulence, amongst others. In addition, it has recently been implicated in the pathophysiology of stunting, a disease that affects millions of children worldwide. Risk factors such as acid-suppressive therapies, alterations in gastrointestinal motility and anatomy, as well as impoverished conditions, have been shown to predispose children to SIBO. SIBO can be diagnosed via culture-dependant or culture-independent approaches. SIBO's epidemiology is limited due to the lack of uniformity and consensus of its diagnostic criteria, as well as the paucity of literature available. Antibiotics remain the first-line treatment option for SIBO, although emerging modalities such as probiotics and diet manipulation could also have a role. Herein, we present a state-of-the-art-review which aims to comprehensively outline the most current information on SIBO in children, with particular emphasis on the gut microbiota.

## Introduction

**Figure 1:**
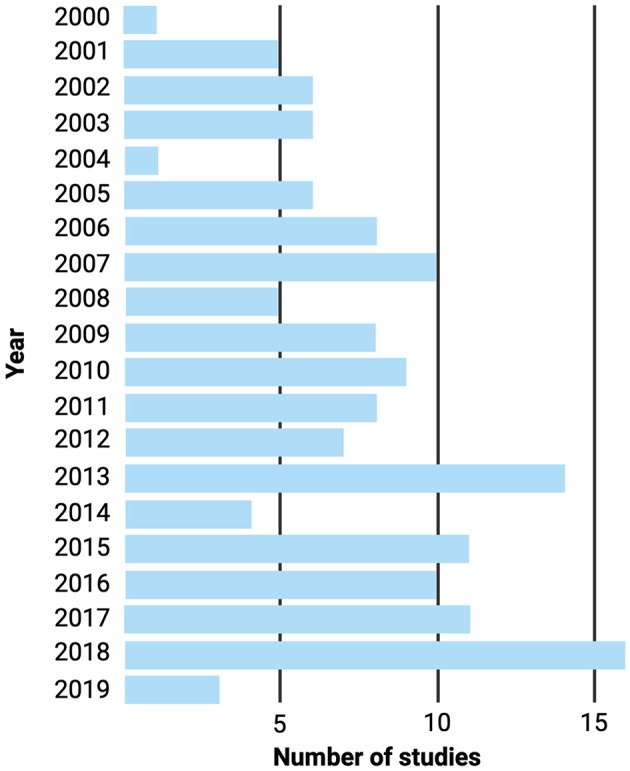
Number of paediatric SIBO publications on PubMed from 2000 to 2019. PubMed literature search with the entry block “(children OR pediatrics) AND (small intestinal bacterial overgrowth OR small intestine bacterial overgrowth OR small bowel bacterial overgrowth)”; the search was conducted on March 26, 2019.

Small intestinal bacterial overgrowth (SIBO) is a heterogenous disorder characterised by an excessive growth of select microorganisms within the small intestine. This excessive bacterial biomass, in turn, disrupts host physiology in a myriad of ways, leading to gastrointestinal and non-gastrointestinal symptoms and complications (1). SIBO is a common cause of non-specific gastrointestinal symptoms in children, such as chronic abdominal pain, abdominal distention, diarrhoea, and flatulence, amongst others (2–5). In addition, it has recently been implicated in the pathophysiology of stunting (6), a disease that affects millions of children worldwide. Certain risk factors, such as acid-suppressive therapies (7–10), alterations in gastrointestinal motility and anatomy (11–20), and impoverished conditions (21–26), have been shown to predispose children to SIBO. Despite the relatively high prevalence of SIBO in children, it remains a poorly understood disorder. In fact, only a small number of studies have been published in the last two decades. Since the year 2000, only 149 articles have been published on paediatric SIBO (Figure 1). Due to this previous scarcity of literature and the recent emergence of novel, informative data regarding the underpinnings of the disease, an update for practicing paediatricians is now warranted. This article represents a state-of-the-art review which aims to comprehensively outline the most current information on SIBO in children, with particular emphasis on the gut microbiota.

## Search Strategy and Selection Criteria

In December 2018, two investigators systematically searched the PubMed/MEDLINE, EBSCOhost, Google Scholar, and ResearchGate databases using the primary search blocks “small intestine bacterial overgrowth OR small intestinal bacterial overgrowth OR small bowel bacterial overgrowth AND children” and “small intestine bacterial overgrowth OR small intestinal bacterial overgrowth OR small bowel bacterial overgrowth.” In addition, the following terms were used in combination: “small intestinal bacterial overgrowth,” “pathophysiology,” “16S rRNA sequencing,” “next generation sequencing,” “gut microbiota,” “proton pump inhibitors,” “juvenile systemic sclerosis,” “scleroderma,” “ileocecal valve,” “vitamin,” “functional gastrointestinal disorders,” “chronic abdominal pain,” “irritable bowel syndrome,” “constipation,” “hydrogen,” “methane,” “breath test,” “stunted,” “environmental enteric dysfunction,” “environmental enteropathy,” “*Methanobrevibacter smithii*,” “methanogens,” “immunodeficiency,” and “coeliac disease.” The search was restricted to titles and abstracts written in English and Spanish, and no date range restriction was applied. We also searched for ongoing clinical trials in Clinicaltrials.gov with the term “small intestinal bacterial overgrowth.” Adult patient studies were included when relevant. In addition, we screened the reference lists of the selected papers and created weekly alerts on PubMed to enhance the sensitivity of the search. The last literature search was conducted on May 13th, 2019.

## The Gastrointestinal Tract and the Gut Microbiota

Each of the organs and anatomical portions of the gastrointestinal system provides specific functions and are equipped with mechanisms to promote a homeostatic relationship with its indigenous bacterial community (microbiota) (27, 28). This mutualistic homeostasis is achieved through mechanisms including gastric and bile acids, pancreatic and digestive secretions, intestinal motility, the barrier function of the ileocecal valve, amongst others. Within the lumen of the gastrointestinal tract resides the gut microbiota, a complex and susceptible microbial consortium composed of enteric-adapted bacteria, which is acquired in the very early stages of life and develops in concert with the host. The “sterile womb hypothesis” refers to the notion that the neonate acquires the gut microbiota during birth through ingestion of the mother's vaginal (vaginal delivery) or skin (caesarean section) microbiota. In contrast, although increasing evidence has suggested that the infant gut microbiota is acquired *in utero*, a technically comprehensive multimodal investigation recently failed to detect any placenta dwelling microorganisms (29). The gut microbiota maintains a life-long symbiotic relationship with the human host, providing a plethora of important functions; from vitamin and short-chain fatty acid (SCFA) production to immunoregulation and neuropeptide secretion [for review see (30)].

**Figure 2:**
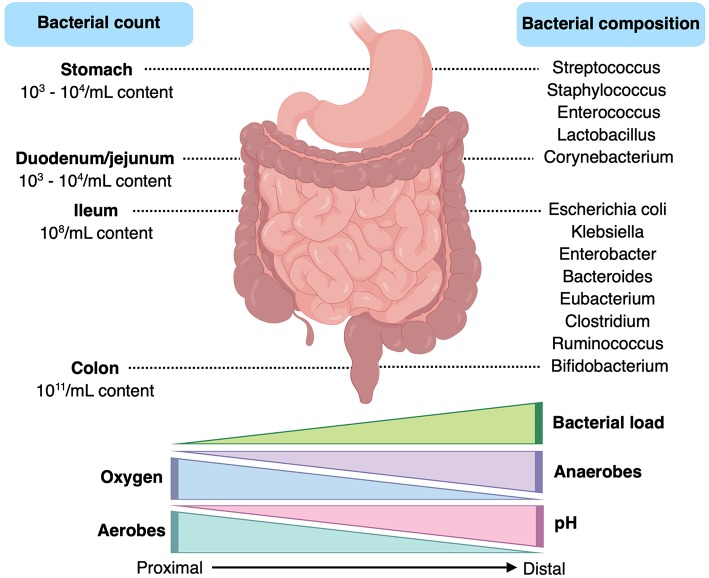
Gastrointestinal tract features and bacterial composition and content. Created with BioRender.com.

The composition and functionality of the gut microbiota is shaped and influenced by a multitude of intrinsic and extrinsic factors, such as genetics, mode of delivery, gestational age, feeding type and diet, pharmaceuticals, and exercise (30). The composition of the gut microbiota exhibits variations related to the anatomical portion studied, which is mainly influenced by factors such as the luminal oxygen concentration and pH. Indeed, bacterial numbers increase from about 10^3^ to 10^4^/ml in the stomach to approximately 10^11^/ml in the colon (Figure 2) (31–33). The colonic microbiota is the largest prokaryotic community in the human body, representing nearly the 0.3% of the overall host body weight (34). Although composed largely of four bacterial phyla –Actinobacteria, Firmicutes, Bacteroidetes, and Proteobacteria– the infantile gut microbiome is dominated by Actinobacteria, with Firmicutes emerging to dominate after infancy (35). In general, bacteria from the Actinobacteria (e.g., *Bifidobacterium*), Firmicutes (e.g., *Faecalibacterium, Clostridium, Ruminococcus, Lactobacillus*) and Bacteroidetes (e.g., *Bacteroides, Prevotella*) phyla are largely regarded as commensal microorganisms, while a significant portion of gastrointestinal pathogens belong to the Proteobacteria phylum (e.g., *Escherichia, Shigella, Salmonella, Klebsiella*, and *Helicobacter*, amongst others) (36, 37). In addition to this diverse community of bacteria, the human gastrointestinal tract is also home to an extensive array of viruses (38), fungi (39), and archaea (40). While it remains unclear at present as to whether the former two kingdoms play a significant role in SIBO pathogenesis, methanogenic archaea, such as *Methanobrevibacter* spp., have been directly implicated in a methane-specific form of the disorder (41).

It is important to note, however, that despite the substantial advances made in the study of the gut microbiota in general, the small bowel microbiota remains poorly understood (42). Whereas the colonic microbiota is more easily accessible and can be sampled via colonoscopy or faecal sample (43), sampling of the small bowel microbiota poses a major challenge due to the invasiveness of the procedures (upper endoscopy) and the technical difficulties associated with these. Therefore, this significantly obscures our understanding of SIBO and hampers the establishment of a definition.

## The Epidemiology of SIBO in Children

The prevalence of SIBO in children has been explored in a wide spectrum of clinical contexts, including children living in impoverished conditions, individuals with chronic abdominal pain (CAP), as well as those who suffer from irritable bowel syndrome (IBS), stunting, and obesity, amongst other diseases. SIBO prevalence ranges from about 9% in children taking proton pump inhibitors (PPIs) (7) to approximately 90% in those with stunted growth (6) and chronic abdominal pain (CAP) (2). However, it should be noted that the data on the epidemiology of SIBO in children is limited by the small number of studies available, the lack of appropriate controls in some studies, and the varying test methodology and diagnostic cut-offs applied. Table 1 shows the study characteristics and reported SIBO prevalence in children with a wide variety of clinical contexts and risk factors.

**Table 1:** Study characteristics and reported SIBO prevalence in children with a wide variety of clinical contexts and risk factors.

**References**	**Year**	**Study population**	**Study design**	**Sample size (n)**	**Diagnostic tests and criteria for positivity**	Reported SIBO prevalence^*^
Pignata et al. (44)	1990	Children with immunodeficiency syndromes aged 2 to 17 years and age-matched control children.	Cross-sectional	Cases: 17 Controls: 10	Jejunal aspirate cultures (not performed in controls due to ethical reasons). Positivity was defined as ≥10^5^ CFU/ml. GHBT Cut-off point was defined as 10 ppm.	Jejunal aspirate: Cases: 41%
Pereira et al. (21)	1991	Children under the age of 5 years living a rural village in Myanmar.	Cross-sectional	Cases: 340	LHBT Positivity was defined as “a transient breath hydrogen peak at the 20, 40, or 60 min breath samples following the lactulose test meal, and distinguishable from the later colonic peak”.	27.2%
de Boissieu et al. (3)	1996	Children with chronic diarrhoea, abdominal pain, or both, aged 2 months to 12 years.	Prospective	Cases: 53	GHBT. Positivity was defined as a H_2_ value ≥10 ppm over baseline after ingestion of glucose.	34%
Lewindon et al. (13)	1998	Children with cystic fibrosis and non-cystic fibrosis children (controls).	Cross-sectional	Cases: 19 Controls: 508	LHBT Positivity was not specified.	Cases: 32% Controls: 7% *P:* <0.003
Fontanele Soares et al. (20)	2005	Children with chronic constipation aged 3 to 13 years.	Cross-sectional	Cases: 40	CH_4_ breath test Positivity (methane producers) was defined as a methane concentration <3 ppm.	73.5%
Dos Reis et al. (22)	2007	Children living in a slum and age and sex-matched controls aged 5 to 11 years.	Cross-sectional	Cases: 50 Controls: 50	Glucose and lactulose H_2_ breath tests. Positivity was defined as an increase in H_2_ of ≥20 ppm over baseline in the initial 60 min.	Lactulose: Cases: 37.5% Controls: 2.1% *P*: <0.001
Fridge et al. (12)	2007	Children and adults with CF and pancreatic insufficiency (mean age 17 years) and age-matched controls.	Cross-sectional	Cases: 25 Controls: 25	Glucose H_2_/CH_4_ breath test. Positivity was defined as either a fasting H_2_ ≥15 ppm, a rise of ≥10 ppm over baseline at any time during the test, or a doubling of baseline CH_4_ excretion at any time during the test. CH_4_ excretors were defined by a CH_4_ level of >2ppm in any sample.	Cases: 56% Controls: 20% *P:* 0.02
Scarpellini et al. (45)	2009	Children with IBS (Rome II criteria) and healthy age- and sex-matched controls.	Cross-sectional	Cases: 43 Controls: 56	Lactulose H_2_/CH_4_ breath test. Positivity was defined as an early rise in H_2_ or CH_4_ excretion of >20 ppm within the first 90 min.	Cases: 65% Controls: 7% *P:* <0.00001
Lisowska et al. (11)	2009	Children with cystic fibrosis and controls with gastrointestinal symptoms aged 5 to 17 years.	Cross-sectional	Cases: 62 Controls: 390	Glucose H_2_/CH_4_ breath test. Positivity was defined as a fasting H_2_ or CH4 level of ≥20 ppm and ≥10 ppm, respectively; or an increase in H_2_ or CH4 over baseline during the test of ≥12 pm and ≥6, respectively.	Cases: 37.1% Controls: 13.3% *P:* <0.00001
Collins et al. (46)	2010	Children with CAP (Rome II criteria) aged 8 to 18 years and healthy controls.	Cross-sectional	Cases: 75 Controls: 40	LHBT. Positivity was defined as a rise in H_2_ >20 ppm before the first 90 min	Cases: 91% Controls: 35% *P:* <0.0001
Cole et al. (16)	2010	Children younger than 2 years with short bowel syndrome receiving enteral feeds	Prospective	Cases: 10	GHBT Positivity was defined as either an increased fasting breath H_2_ ≥ 20 ppm or increase from baseline of ≥10 ppm after glucose ingestion	(incidence) 50%
Leiby et al. (15)	2010	Children with secondary retentive faecal incontinence (and radiographically diagnosed faecal impaction) aged 6 to 12 years and controls with gastrointestinal symptoms but without faecal incontience.	Cross-sectional	Cases: 50 Controls: 39	Lactulose H_2_/CH_4_ breath test. [n] Positivity was defined as an increase in H_2_ ≥20 ppm or in CH_4_ ≥10 ppm over baseline at ≤ 60 min. Patients were considered CH_4_ producers if their level was more than 3 ppm at any point in the study; and high basal CH_4_ was diagnosed if the baseline sample was >10 ppm.	Cases: 42% Controls: 23%
Jones et al. (47)	2011	Children with chronic diarrhoea and/or abdominal pain and/or bloating and/or irritability younger than 15 years of age.	Cross-sectional	Cases: 287	CO2-corrected H_2_ and CH_4_ levels Positivity was defined as an increase in H_2_ >10 ppm over baseline in the initial 45 min of the test. Patients were classified as H_2_ or CH_4_ producers if they produced >10 ppm of these gases at any time point.	87%
Mello et al. (23)	2012	Children of poor socioeconomic conditions residing in a slum and children of socioeconomically advantaged families aged 6 to 10 years.	Cross-sectional	Cases: 85 Controls: 43	Lactulose H_2_/CH_4_ breath test. Positivity was defined as an increase in H_2_ of ≥ 20 ppm or CH4 of ≥ 10 ppm with respect to the fasting value within the first 60 min after the ingestion of lactulose? Subjects were considered CH_4_-producers when the concentration of CH_4_ was ≥ 3.	Cases: 30.9% Controls: 2.4% *P:* 0.0007
Gutierrez et al. (17)	2012	Children with intestinal failure and refractory gastrointestinal symptoms (i.e., abdominal bloating, emesis, diarrhoea, or increased stoma output) with a median age of 5 years.	Cross-sectional	Cases: 57	Duodenal aspirate cultures. Positivity was defined as a bacterial growth of ≥10^5^ CFU/ml.	70%
Scarpellini et al. (48)	2013	Children with IBS (Rome II criteria). *Study objective:* to assess the effects of rifaximin treatment on SIBO prevalence and gastrointestinal symptoms in children affected by IBS.	Prospective	Cases: 50	Lactulose H_2_/CH_4_ breath test. Positivity was defined as an early rise in H_2_ or CH_4_ excretion >20 ppm within the first 90 min.	66%
Ojetti et al. (14)	2013	Children with myelomeningocele and constipation.	Cross-sectional	Cases: 18	Lactulose H_2_/CH_4_ breath test. Positivity was not specified.	39%
Hegar et al. (9)	2013	Children with epigastric pain and a normal baseline GHBT aged ≥5 years were divided into two 4-week trial groups: group 1 (cases): omeprazole plus Lacidofil® (1.9 ×109 CFU *Lactobacillus rhamnosus* R0011 and 0.1 ×109 CFU *Lactobacillus acidophillus* R0052); and group 2 (controls): omeprazole plus placebo capsule. *Study objective:* to evaluate the incidence of SIBO in children treated with omeprazole and to test whether probiotics influence the incidence.	Double-blinded, placebo-controlled randomized clinical trial	Cases: 36 Controls: 34	GHBT. Positive test was defined as an increase in H_2_ of >10 ppm over baseline.	(incidence) Cases: 33% Controls: 26% *P:* 0.13
Rosen et al. (8)	2014	Children taking acid suppressive therapy for a minimum of 4 weeks (PPIs and histamine-2 antagonists) and controls (no acid suppressive therapy) aged 1 to 18 years	Cross-sectional	Cases: 48 Controls: 51	Gastric aspirate cultures. Positivity was not clearly specified.	Cases: 46% Controls: 18% *P: 0.003*
Korterink et al. (4)	2014	Children with abdominal pain–related functional gastrointestinal disorders (AP-FGID; Rome III criteria) aged 6 to 18 years.	Prospective	Cases: 161	GHBT. Positivity was defined as fasting breath H_2_ concentration ≥20 ppm or increase in H_2_ ≥12 ppm over baseline value.	14.3%
Lisowska et al. (49)	2014	Children with progressive familial intrahepatic cholestasis aged 8 to 25 years.	Prospective	Cases: 26	Glucose H_2_/CH_4_ breath test. Positivity was defined as a high baseline value (>20 ppm for hydrogen or >10 ppm for methane) or an early increase of gas excretion (>12 ppm for hydrogen or >6 ppm for methane).	35%
Sieczkowska et al. (10)	2015	Children with histology-proven peptic esophagitis aged 3 to 18 years. *Study objective*: to evaluate whether a 3-month PPI treatment regimen induces SBBO in children and if so, to determine associated symptoms.	Prospective	Cases: 40	Glucose H_2_/CH_4_ breath test. Positivity was defined as an increase in H_2_ of ≥10 over baseline.	22.5%
Donowitz et al. (25)	2016	Bangladeshi children from an impoverished neighbourhood aged 2 years.	Cross-sectional	Cases: 90	GHBT. Positivity was defined as an increase in H_2_ of ≥12 ppm over baseline.	16.7%
Cares et al. (7)	2017	Children taking PPIs for longer than 6 months' duration and controls (no PPI treatment) aged 3 to 17 years. *Study objective:* to measure the risk for SIBO in children on chronic PPI therapy and compare, using the glucose HBT, the frequency of SIBO in children taking PPIs with those who did not.	Cross-sectional	Cases: 56 Controls: 27	Glucose H_2_/CH_4_ breath test. Positivity was defined as an increase in either H_2_ or CH_4_ of >12 ppm over baseline.	Cases: 8.9% Controls: 3.7% *P:* 0.359
Wang et al. (50)	2017	Children with autism spectrum disorder and age- and sex-matched healthy controls (age was not specified)	Cross-sectional	Cases: 310 Controls: 1240	Glucose H_2_/CH_4_ breath test. Positivity was defined as an increase in H_2_ of ≥20 ppm or CH_4_ of ≥10 ppm over baseline before the first 60 min.	Cases: 31.0% Controls: 9.3% *P: <* 0.0001
Mello et al. (24)	2017	Children of low socio-economic status living in an urban slum aged 5 to 11 years	Cross-sectional	Cases: 100	Lactulose H_2_/CH_4_ breath test. Positivity was defined as an increase in H_2_ of ≥20 ppm or CH_4_ of ≥10 ppm over baseline before the first 60 min. Real-time polymerase chain reaction (results are described in aetiology section)	61.0%
Belei et al. (51)	2017	Children with overweight or obesity aged 10 to 18 years and age- and sex-matched controls	Cross-sectional	Cases: 125 Controls: 120	GHBT Positivity was defined as an increase in H_2_ in two consecutive measurements of at least 15 ppm over baseline.	Cases: 37.6% Controls: 3.3%
Galloway et al. (18)	2018	Children with intestinal failure aged 9 months to 17 years.	Prospective	Cases: 14	Duodenal aspirate culture. Positivity was defined as ≥10^5^ CFU/ml.	43%
Gaffar et al. (26)	2018	Children living in a disadvantaged urban community aged 12 to 18 months.	Prospective	Cases: 194	GHBT Positivity was defined as an increase in H_2_ of ≥12 ppm over baseline measurement on any single post-glucose reading.	14.9%
Furnari et al. (52)	2018	Subjects with cystic fibrosis older than 2 years.	Randomised, case-control Trial	Cases: 79	Glucose H_2_/CH_4_ breath test. Positivity was defined as either a H_2_ basal level of ≥12 ppm, a peak of H_2_ excretion of ≥10 ppm over baseline during the test, or a CH_4_ level of ≥12 ppm at any point time.	31.6%
Vonaesch et al. (6)	2018	Stunted children aged 2 to 5 years and healthy controls.	Cross-sectional	Cases: 46	Duodenal aspirate culture. Positivity was defined as ≥10^5^ CFU/ml. 16S rRNA sequencing (results are described in the text)	Cases: 96%

* *A few studies also evaluated the incidence of SIBO in children (indicated in parenthesis). GHBT, glucose hydrogen breath test; LHBT, lactulose hydrogen breath test; H_2_, hydrogen; CH_4_, methane; CO_2_, carbon dioxide; ppm, parts per million*.

## SIBO Pathogenesis

**Figure 3:**
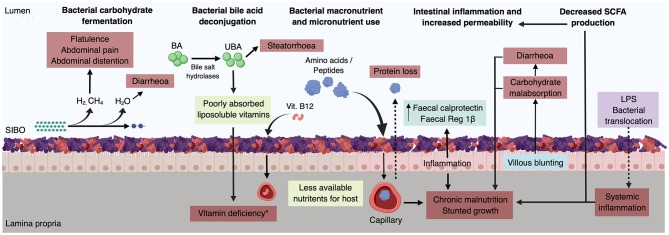
Mechanisms through which SIBO affects the host. The dotted arrows indicate increased intestinal permeability. ^*^ Includes vitamin A, D, E, and vitamin B 12. Vitamin K is synthesised by the gut microbiota, and thus its deficiency in this context is very unlikely. BA, bile acids; UBA, unconjugated bile acids; LPS, liposaccharides. Created with BioRender.com.

SIBO can negatively impact the host in a range of ways (1, 31, 53–61). These include bacterial carbohydrate fermentation leading to excess gas and water production (1); bacterial deconjugation of bile acids resulting in poorly absorbed liposoluble vitamins (18); bacterial macronutrient and micronutrient consumption (bacterial-host nutrient competition), leaving the host with less available nutrients for absorption (58); villous blunting leading to carbohydrate malabsorption (13, 62–64); decreased short chain fatty acid production (6, 65); intestinal and systemic inflammation (16, 25, 66); and increased gut permeability (Figure 3). It should be noted that a recent systematic review (53) and clinical study (25) found conflicting and contradictory evidence supporting effects of gut permeability.

## Risk Factors for SIBO

### Acid-Suppressive Therapies

Gastric acid plays a crucial role in preventing pathogens from colonizing the human alimentary tract, particularly the proximal portions (67). Several extrinsic and intrinsic factors are known to alter this natural barrier (68), with one of them being medication-induced hypochloridria, caused most frequently by proton pump inhibitors (PPIs). PPIs are commonly prescribed for the treatment of gastroesophageal reflux disease (GERD) and, sometimes, for gastroesophageal reflux (GER) in children (69). These drugs decrease gastric acid secretion by blocking the enzyme H^+^/K^+^-adenosine triphosphatase located in the apical membrane of parietal cells (70). It has been estimated that approximately 34% (69) of paediatric patients treated with PPIs develop adverse effects, including infectious diarrhoea, *Clostridium difficile* infection, respiratory infections, or SIBO (71–77). In addition, PPIs have also been shown to alter the faecal gut microbiota of children and adults (78, 79). In theory, the resultant acid suppression creates a more favourable environment for bacteria to overgrow –particularly Gram-positive, aerobic bacteria (31)–, leading to the development of SIBO. However, a recent observational study (6) found contradictory evidence: despite the high prevalence of SIBO seen in the patient cohort (i.e., 96%), gastric pH was found to be in the acidic range (i.e., pH 2.7). It is important to note that pH measurements were only obtained from the study group and lacked normal reference values for comparison.

To date, four studies have assessed SIBO risk in children taking acid-suppressive treatments (80). Cares et al. (7) used the glucose H_2_/CH_4_ breath test to determine the presence of SIBO in children taking PPIs for a prolonged time (i.e., longer than 6 months). 77% of patients took a PPI for over 12 months, and SIBO was diagnosed in 8.9% and 3.7% of PPI-subjects and controls, respectively. Moreover, Sieczkowska et al. (10) enrolled children with histology-proven peptic esophagitis in a 3-month trial of omeprazole treatment. A glucose hydrogen breath test (GHBT) was performed before and after PPI treatment, revealing that 22.5% developed SIBO as a result of the therapy. Furthermore, Rosen et al. (8) cultured the gastric fluid of children taking PPIs for at least 4 weeks and whose last dose was taken within 24 h of sampling. Compared to the control group, the PPI-group was found to have a significantly higher prevalence of gastric overgrowth (18 vs. 46%, respectively), mainly caused by potential pathogens such as *Staphylococcus* and *Streptococcus*. In a double-blind, placebo-controlled randomised clinical trial, Hegar et al. (9) randomly assigned children to one of two 4-week treatment groups: omeprazole + placebo group or omeprazole + probiotic. SIBO had been excluded at baseline in all. Following the 4-week intervention, a second GHBT indicated that SIBO was present in 33% and 26% of omeprazole/probiotic group and omeprazole/placebo group, respectively.

Two metanalyses (81, 82) of adult patients evaluated the association between PPI therapy and SIBO risk. The most recent study by Su et al. (81) included 19 observational studies with a total of 7,055 adult subjects in the analysis. Although there was a significant degree of heterogeneity amongst the studies included, after adjusting for study quality, the authors concluded that PPI therapy was associated with a moderately increased risk for SIBO (odds ratio 1.71, 95% CI 1.20–2.43). The second meta-analysis (83) included 11 studies [all of which were included in the metanalysis by Su et al. (81)] with a total of 3,134 adult subjects. Again, the authors found a statistically significant association (odds ratio, 2.282, 95% CI 1.238–4.205) between PPI therapy and SIBO, but only when diagnosis was made with “highly accurate testing modality,” such as duodenal/jejunal aspirate culture. It is important to mention, however, that all the studies included in both metanalyses were observational in nature and culture or breath test-based approaches were used for SIBO diagnosis.

In conclusion, it appears that acid suppressive therapies, and in particular PPIs, seem to be a risk factor for SIBO development, but only when this is assessed by culture approaches. However, it is important to mention that the data are limited by the overall low odds ratios (~2) and the lack of controls. Thus, until better data are available, it is of paramount importance that PPI prescriptions in children with possible GERD are judicious, and perhaps more importantly, that their use is avoided in children with GER (72, 83).

### Intestinal Motility Disturbances

In addition to the acidic environment created by the gastric acid, gut motility is critical to preventing SIBO. One motor event, the migrating motor complex (MMC), a cyclic motor pattern that occurs during the interdigestive state, plays an important role in preventing the development of bacterial overgrowth within the small intestine (83). In fact, the MMC is commonly referred to as the “intestinal housekeeper,” emphasising its role in gastrointestinal health (84). Under physiological conditions, the MMC sustains the aboral progression of luminal content in the small intestine between meals, thereby preventing stasis and SIBO. On this basis, an absent or disrupted MMC may lead to bacterial overgrowth (84).

The typical example of a disease with gut dysmotility leading to SIBO is systemic sclerosis, with ~40% of adult patients being affected (54). Despite the relatively high prevalence of gastrointestinal symptoms in children with juvenile systemic sclerosis (jSS) (85), to our knowledge, no studies have investigated the association of SIBO with jSS.

A somewhat more common example of gut dysmotility associated with increased risk of SIBO is cystic fibrosis (CF) (86). Here there is limited clinical data from studies in children. Lisowska et al. (11) found a significantly higher SIBO prevalence in patients with CF compared with non-CF subjects (37 vs. 13%, respectively), and so did Fridge et al. (12) who found a significantly higher prevalence in CF-subjects than controls (56 vs. 20%, respectively). Both studies used the hydrogen breath test (HBT) for SIBO diagnosis. Moreover, Lewindon et al. (13) evaluated, with the HBT, SIBO prevalence and orocecal transit time of children with CF. Interestingly, compared with the two control groups (i.e., healthy children and non-CF patients), CF-subjects had a significantly higher SIBO prevalence and their orocecal transit times were significantly longer than those of healthy individuals. More recently, Furnari et al. (52) found a 31% SIBO prevalence in both children and adults with CF. Preclinical studies in murine models have demonstrated that CF-mice have a higher SIBO prevalence than wild-type mice (87), which is thought to be mainly caused by two factors: (1) slowed intestinal transit –possibly due to unabsorbed lipids leading to a triggering of the “ileal brake”– and/or smooth muscle dysfunction (86, 88); and (2) mucus accumulation which acts as an anchor for bacteria, thereby facilitating their overgrowth (87, 89). Thus, the multifactorial nature of CF, including gut dysmotility and impaired mucus clearance, seems to put patients at a higher risk of SIBO development.

Constipation has been shown to be associated with SIBO. However, the causative or consequential nature of this interaction is unclear. In theory, a slower orocecal transit in constipation may fail to clear the luminal content, thereby increasing SIBO risk. On the other hand, methane, a biologically active gas produced instead of hydrogen by some individuals from bacterial fermentation of carbohydrates, can delay intestinal transit, which in turn may lead to constipation. We identified three studies that investigated the role of SIBO in children with constipation. Ojetti et al. (14) diagnosed SIBO in 39% (7/18) of children with myelomeningocele, a disease associated with constipation. Interestingly, the authors found that all methane producers had a delayed orocecal transit time. Moreover, Leiby et al. (15) found a 42% (21/50) SIBO prevalence in children with retentive faecal incontinence, of whom, eight had methanogenic SIBO, 11 had hydrogen-type SIBO, and two had mixed (methane and hydrogen)-type SIBO. In addition, 48% of patients with faecal incontience were found to have high basal methane concentrations (>10 ppm) as compared with 10% of control subjects. Furthermore, Fontanele Soares et al. (20) investigated the relationship between methane production and colonic transit time in children with constipation. Methane production was found in 73.5% (25/34) of children with constipation and soiling as compared with 1% of children with constipation alone.

The notion that a slowed intestinal transit may predispose to SIBO is supported by two recent studies in adults (90, 91). In the most recent study, by Revaiah et al. (90), two patient groups (i.e., PPI-group and PPI + prokinetic group) underwent a GHBT and lactulose hydrogen breath test (LHBT) and orocecal transit time assessment. Interestingly, SIBO was documented more frequently in the PPI alone group than in the PPI + prokinetic group, and SIBO-positive patients had slower orocecal transit times than SIBO-negative patients. However, it is important to note that the overall SIBO prevalence was only 8.8% (13 subjects) and, even though the authors measured both hydrogen and methane, it was not specified whether the three patients with methane-positive SIBO had normal or delayed orocecal transit times. Furthermore, Sarosiek et al. (91) prospectively enrolled 29 female patients with functional constipation in a 2-week lubiprostone trial. Gastrointestinal transit and SIBO were assessed by the wireless motility capsule (WMC) and lactulose hydrogen/methane breath tests, respectively. At baseline, 68% of patients had increased levels of both hydrogen and methane, suggesting SIBO. After treatment, 41% of patients became SIBO-negative, which was paralleled by a 30% increase in small bowel transit time. However, it is important to mention that the authors considered methane positivity as an increase of ≥3 ppm, which differs from the current guidelines in which methane positivity is defined as an increase of ≥10 ppm (92). These findings are difficult to interpret for two reasons: (1) all patients had constipation and were methane producers at baseline (i.e., methane production of ≥3 ppm); thus, this suggests constipation may have been caused by intestinal methane production rather than constipation leading to SIBO; or simply, the methane values present in these patients could be physiological; and (2), the fact that lubiprostone “cured” 41% of SIBO patients suggests that by increasing the intestinal transit–and thus intraluminal clearance–faecal stasis decreases, which prevents SIBO development.

### Anatomical Alterations

Anatomical boundaries are crucial for maintaining a harmonized microbial community within the human alimentary tract, particularly the integrity of the ileocecal valve. Absence or dysfunction of the ileocecal valve has been shown to predispose patients to SIBO, as colonic bacteria are thought to be “backwashed” into the small intestine, thereby leading to coliform-type SIBO development (31).

Paediatric intestinal failure is a disease characterised by increased SIBO prevalence, which is thought to be due to a multifactorial process that includes altered intestinal motility and anatomy, resection of the ileocecal valve, and use of antacids (93, 94). Only four recent studies have investigated SIBO incidence/prevalence in children with intestinal failure (or short bowel syndrome) (16–19). The most recent study by Hong et al. (19) found a 78% SIBO prevalence in their patient cohort of children who underwent extensive small bowel resections due to midgut volvulus. Interestingly, only 22% of patients had a functional ileocecal valve. Moreover, Galloway et al. (18) diagnosed SIBO by duodenal aspirate cultures in 43% of patients with intestinal failure, caused mainly by gastroschisis, atresias, and necrotizing enterocolitis. Of these, only one subject had an ileocecal valve (16%). Furthermore, Gutierrez et al. (17) diagnosed SIBO in 70% of patients with intestinal failure due to various surgical and non-surgical aetiologies. Although the authors did not find a significant difference regarding SIBO prevalence between children with and without ileocecal valve, they did find a significant association between parenteral nutrition (PN) use and SIBO. The fourth study by Cole et al. (16) found a 50% SIBO incidence in their patient cohort of children with short bowel syndrome caused by necrotizing enterocolitis. Of these, 40% of children had a functional ileocecal valve. In line with the findings by Gutierrez et al. (17), another interesting study (95) that evaluated the faecal gut microbiota of children with short bowel syndrome by next generation sequencing found that children who were on PN had decreased diversity and increased numbers of Proteobacteria as compared with those who were weaned from PN. None of the PN-patients had an ileocecal valve and, of these, four out five patients were being evaluated for SIBO and were being treated with antibiotics, which may explain the microbiota perturbations observed.

Taken together, these findings demonstrate that paediatric intestinal failure is a multifactorial process that increases the risk of SIBO, as these children have a disrupted intestinal anatomy, physiology, and microbiota which is frequently exposed to antacids and antibiotics. Resection of the ileocecal valve, in particular, appears to be a common finding in children with intestinal failure and SIBO, strongly suggesting the role of this structure in SIBO prevention. In line with this, Roland et al. (96) investigated the ileocecal junction pressure by WMC and the presence of SIBO by the LHBT and small bowel aspirate cultures. Interestingly, the authors found a combination of SIBO-predisposing factors in their patient cohort: (1) the small bowel transit time in SIBO-patients was significantly slower that those without SIBO; (2) the gastric pH was significantly higher in SIBO-patients than those without SIBO; and (3) the mean ileocecal junction pressure was significantly lower among SIBO-patients than those without SIBO. Thus, these findings reinforce the fact that an “incompetent” ileocecal valve predisposes to SIBO and emphasise the multifactorial nature of the disease.

### Impoverished Conditions and Poor Socioeconomic Status

Impoverished conditions, which are commonly associated with a lack of basic sanitation services such as clean water, appropriate sewage, and collection of household garbage, may be a risk factor for SIBO. To our knowledge, six studies [one from Myanmar (21), three from Brazil (22–24), and two from Bangladesh (25, 26)] have investigated this association in the paediatric population.

Pereira et al. (21) investigated the prevalence of SIBO in children from a Burmese village (Myanmar) by using the LHBT. Around 85% of the village's population obtained drinking water from surface wells and ponds, and approximately 10% used rain water for the same purposes (97). The authors diagnosed SIBO in 27% of cases (53/195), with males being more commonly affected than females. Moreover, dos Reis et al. (22) found a significantly higher SIBO prevalence in children living in a slum as compared with controls living in households with appropriate sanitation services (37.5 vs. 2.1%, respectively). In addition, Mello et al. (23) also found a significantly higher SIBO prevalence in the slum-group compared with their socioeconomically advantaged counterparts (30.9 vs. 2.4%, respectively); however, it is important to note that the authors did not find statistically significant differences in the environmental variables (i.e., water contamination with coliforms, access to public water network, access to public sewage, and public collection of household garbage) between the SIBO-positive and SIBO-negative slum subgroups. The second and more recent study by Mello et al. (24) assessed SIBO prevalence and analysed the faecal gut microbiota of children residing in a Brazilian slum. Roughly 60% of children were found to have SIBO, and their faecal gut microbiota analyses showed a significantly higher number of *Salmonella* spp. and lower numbers of Firmicutes. Donowitz et al. (25), who investigated SIBO prevalence in 90 Bangladeshi children belonging to the lowest socioeconomic strata, reported that 16.7% had a positive GHBT and that the odds of developing SIBO were increased by the presence of an open drain/sewer outside the home (odds ratio, 4.78; 95% confidence interval, 1.06 to 21.62). More recently, Gaffar et al. (26) conducted a prospective nutritional intervention study on stunted and at-risk-of-stunting Bangladeshi children living in an urban slum (98), and found SIBO in 14.9% of subjects.

Taken together, these findings demonstrate that poor sanitation conditions, particularly contaminated water exposure, may expose children to a higher risk of developing SIBO. However, none of these studies provided a clear pathophysiological mechanism by which such unsanitary conditions predispose children to the disease. Donowitz et al. (55) proposed a mechanism of SIBO development in the setting of poor sanitary living conditions. The authors hypothesised that repeated exposure to abnormal levels of liposaccharides found in soil and drinking water may disrupt the MMC, causing faecal stasis (as seen in patients with gut dysmotility), thereby leading to SIBO development. While this theory appears to have some biological plausibility, to date, no studies have evaluated it.

### Other SIBO Risk Factors

Immunodeficiency and coeliac disease (CD) have also been regarded as potential risk factors for SIBO development. As for immunodeficiency, we only identified one small-scale study (44) from 1990, which was conducted on children with immunodeficiency syndromes (i.e., IgA deficiency, hypogammaglobulinemia, and T cell defects). The most common clinical manifestation was chronic diarrhoea and SIBO was diagnosed in 41% of patients via jejunal aspirate culture. Moreover, in a more recent study (99), 4% (*n* = 12/296) of young adults with chronic diarrhoea and malabsorption syndrome were found to have hypogammaglobulinemia. Of these, 25% (*n* = 3/12) were diagnosed with SIBO. Although these findings show a relatively high SIBO prevalence in children and young adults with immunodeficiency syndromes, recent, large-scale studies are needed in order to support this association. As for CD, a recent systematic review (100) of adult patient studies found a high SIBO prevalence in patients with CD (20% pooled mean prevalence). The authors concluded that SIBO may be more common in patients with CD when symptoms do not improve after a gluten-free diet. We identified only two studies (101, 102) that characterized the microbiota composition and diversity of children with CD: subjects with active CD were found to have a higher abundance of members of the phylum Proteobacteria (101) and lower ecological indexes of genus *Lactobacillus* (102) as compared with healthy and non-active CD patients. Although these studies did not set out to evaluate SIBO *per se* and as such did not report CFU/g, the higher Proteobacteria abundance and lower ecological indexes of genus *Lactobacillus* seen in children with CD may indicate a disturbed microbial ecosystem. Certainly, a study that evaluates the presence of SIBO in coeliac children, either by the H_2_/CH_4_ breath test or small intestinal aspirate culture, would be of great interest.

It is important to note, however, that SIBO can develop even in the absence of any of the aforementioned risk factors. In line with the findings by Boissieu et al. (3) and our own experience, many children who test positive for SIBO are “healthy” and have no evident risk factors. There are clearly many questions to be addressed by future studies regarding the risk factors for SIBO in children.

## Aetiology of Paediatric and Adult SIBO

SIBO can be caused by archaea or bacteria, by one or more microorganisms, by Gram-positive or Gram-negative bacteria, and by anaerobic or aerobic microorganisms (31). Pistiki et al. (103) conducted a cross-sectional study in which patients underwent duodenal aspirate cultures for the diagnosis of SIBO with a diagnostic threshold of >10^3^ CFU/mL. SIBO was diagnosed in 20% of cases, being caused by one microorganism in 54.7% of cases and by two microorganisms in the remainder. In addition, the vast majority of bacterial isolates belonged to the phyla Proteobacteria and Firmicutes, with the most common being *Escherichia coli, Enterobacter* spp., *Klebsiella* spp., *Pseudomonas aeruginosa, Staphylococcus aureus, Acinetobacter baumannii, Stenotrophomonas maltophilia, Citrobacter freundii, Serratia marcescens*, and *Enterococcus faecium* (in descending order of frequency). In turn, Pyleris et al. (104) conducted a study with the same diagnostic methodology, finding SIBO in 19.4% of adult subjects. Likewise, most bacterial isolates were members of the Proteobacteria and Firmicutes phyla (*Escherichia coli, Enterococcus* spp. *Klebsiella pneumoniae, Proteus mirabilis, Acinetobacter baumannii, Citrobacter freundii*, and *Serratia marscecens*), and in 75% of cases the overgrowth was due to one microorganism. In the remainder of cases, SIBO was caused by two microorganisms, with the most common dyads being *E. coli* + *K. pneumoniae* and *E. coli* + *Enterococcus* spp. Furthermore, Gutierrez (17) conducted a retrospective study on children with intestinal failure who underwent duodenal aspirate cultures. SIBO was diagnosed in 70% of cases by using a more stringent diagnostic threshold of >10^5^ CFU/mL. Again, the most common causative microorganisms were members of the Proteobacteria and Firmicutes phyla, with the most common being *E. coli, Streptococcus viridans, K pneumoniae, Enterococcus* spp., and *Pseudomonas aeruginosa*. The overgrowth was due to more than one bacterium in a minority of patients. Moreover, Galloway (18) diagnosed SIBO in 43% of children with intestinal failure, of whom five out of six patients had overgrowth due to two different microorganisms and only one had overgrowth caused by one microorganism, with *Enterococcus* and *Klebsiella* being the most frequently isolated bacteria.

More recently, Ghoshal et al. (105) conducted a cross-sectional study on adult subjects with non-alcoholic steatohepatitis (NASH) who underwent a GHBT coupled with jejunal aspirate cultures. The authors defined SIBO as a bacterial growth of ≥10^5^ CFU/mL and low-grade bacterial overgrowth as ≥10^3^ CFU/mL; 20 and 60% of cases were diagnosed with SIBO and low-grade bacterial overgrowth, respectively. Amongst the SIBO cases, Proteobacteria (*Pseudomonas aeruginosa, Klebsiella pneumonia*, and *Acinetobacter* spp.) were the most commonly isolated microorganisms followed by members of the Firmicutes phylum (*Streptococcus* spp. and *Enterococcus faecalis*); 20% of cases were due to overgrowth of more than one microorganism.

Taken together, these findings demonstrate that in most cases, SIBO is caused by a single microorganism that belongs to the Proteobacteria phylum (37), particularly coliform bacteria such as *E. coli* and *Klebsiella spp*. However, it is important to consider three aspects: (1) culture-dependant approaches were used in these studies, which means that there was a risk of missing bacteria that remain difficult to culture under clinical laboratory conditions; (2) only two studies were conducted on children (17), and (3) SIBO was studied in a wide spectrum of clinical contexts, such as IBS, intestinal failure, and non-alcoholic steatohepatitis. Thus, the specificity of such microbiota alterations for SIBO must be interpreted in the context of these potentially important confounders.

Next generation sequencing (NGS) methods such as 16S ribosomal RNA (rRNA) sequencing have become a high-resolution and relatively cost-effective way to study the human gut microbiome (106–108). To our knowledge, only one study in children (6) has used these methods to evaluate the gut microbiota in the context of SIBO. Vonaesch et al. (6) conducted a novel, cross-sectional study on stunted and healthy children from Madagascar and Central African Republic (CAR), in which 16S rRNA amplicon sequencing was used to analyse the faecal gut microbiota composition of both groups and to confirm the presence of bacteria identified by gastric and duodenal fluid aspirate cultures—these were only obtained from the stunted group due to ethical reasons. The authors used the higher diagnostic threshold for SIBO diagnosis (i.e., >10^5^ CFU/mL). Despite this, SIBO was diagnosed in 96% of stunted children (Madagascar: 100%; and CAR: 88%) and, interestingly, the most common causative microorganisms were oropharyngeal colonizers, such as *Streptococcus* spp., *Staphylococcus* spp., *Haemophilus* spp., *Moraxella* spp., and *Neisseria* spp. In addition, microbiota sequencing showed overrepresentation of oropharyngeal species (e.g., *Streptococcus* spp., *Haemophilus* spp., *Neisseria* spp., *Rothia* spp., *Actinomyces* spp., and *Gemella* spp.) and enteropathogens (e.g., *Escherichia coli, Shigella*, and *Campylobacter*), as well as underrepresentation of butyrate producers (e.g., *Clostridia* spp.) in stunted children compared with controls. In contrast with the studies described above (17, 103–105), however, most bacterial overgrowths were caused by members of the Firmicutes phylum followed by the Proteobacteria phylum.

## Clinical Features and Complications of SIBO in Children

Paediatric SIBO is a heterogenous disorder that manifests itself through non-specific symptomatology, including gastrointestinal and non-gastrointestinal symptoms (31, 109). The most common signs and symptoms reported in the literature are chronic abdominal pain, abdominal distention, diarrhoea, flatulence, belching, steatorrhea, fetid stools, mucus in stools, fatigue, nausea, and stunted growth (Table 1) (2–6).

### SIBO and Functional Gastrointestinal Disorders

It is always challenging to discuss functional gastrointestinal disorders (FGIDs) and their possible interaction with an organic aetiology, such as SIBO. A small number of observational studies have evaluated the association between FGIDs and SIBO in the paediatric population.

Korterink et al. (4) determined the presence of SIBO in children with abdominal pain–related functional gastrointestinal disorders [i.e., irritable bowel syndrome (IBS), functional abdominal pain (FAP), functional dyspepsia (FD), and FAP syndrome] by using the GHBT. Amongst the 14.3% SIBO-positive subjects, the most common symptoms were fatigue (75%), altered defecation pattern (71%), nausea (68%), and bloating (66%). However, only altered defecation pattern, loss of appetite, and belching were significantly higher than SIBO-negative subjects; diarrhoea and flatulence did not reach statistical significance. Furthermore, Collins et al. (46) also used the hydrogen/methane breath test to diagnose SIBO in their cohort of children with chronic abdominal pain (i.e., FD, IBS, and FAP). Ninety one percent of cases and 35% of healthy controls had a positive breath test, respectively. Surprisingly, there were no differences in the presence of gastrointestinal symptoms such as bloating, gas, incomplete evacuation, constipation, diarrhoea, mucous in stool, or straining, between SIBO-positive and SIBO-negative subjects; however, it is important to mention that the comparison was disproportional between the two groups (68 vs. 7 patients, respectively). Moreover, Scarpellini et al. (45) evaluated the prevalence of SIBO in children with IBS by the lactulose hydrogen/methane breath test, finding a higher prevalence of SIBO amongst IBS sufferers compared to their healthy counterparts (65 vs. 7%, respectively). Taken together, these findings demonstrate that SIBO is a frequent underlying diagnosis in children with functional abdominal pain disorders (i.e., IBS and FD), thus suggesting a role in their pathogenesis.

The IBS-SIBO interaction has attracted a tremendous amount of research in the last decade, and thus it deserves special attention. A recent systematic review and metanalysis (110) of observational studies in adults estimated the prevalence and determined predictors of SIBO in IBS. The authors found an overall pooled SIBO prevalence of 38% (95% CI 32–44) as well as a 4.7 (95% CI 3.1–7.2) pooled OR of SIBO in IBS subjects as compared with healthy controls. Surprisingly, PPI use was not associated with SIBO. Even though a growing body of literature shows a clear association between the two disorders, it is unclear whether SIBO precedes IBS or vice versa (111, 112). Thus, until better data are available, children presenting with IBS-like symptomatology may merit SIBO-diagnostic workup, such as a H_2_/CH_4_ breath test [SIBO's role in IBS is comprehensively reviewed elsewhere (61, 113)].

Constipation, another FGID, has been associated with intestinal methane production in adults and children (methanogenic SIBO) (114) Indeed, a 2011 metanalysis (115) of nine studies (1,277 subjects) found a significant association between methane on breath test and constipation (OR 3.51, CI 2.00–6.16). Moreover, Pimentel et al. (116) demonstrated this association in animal and human models: intraluminal infusion of methane reduced small bowel transit by 59% in canine models as compared with controls, and methane was also found to increase intestinal contractile activity in guinea pigs and in patients with IBS. In contrast, Mello et al. (23) did not find an association between methane and constipation in their patient cohort of children living in a slum; in fact, none of these children had constipation despite a 30% prevalence of methanogenic SIBO. More recently, Ghoshal et al. (117) investigated the effect of rifaximin on breath methane and colonic transit in adult patients with constipation. The authors found that a larger percentage of patients with chronic constipation were methane producers (>10 ppm) and had slower colonic transit times as compared with controls. Methane producers (*n* = 13) were randomly assigned into two groups: rifaximin group (14-day trial) and placebo group. After treatment, the rifaximin group had a significantly lower area under the curve for methane production compared with the placebo group, and colonic transit time normalized in 66% of cases as compared with the placebo group, in whom colonic transit time never normalized. Thus, these findings support the association between methanogenic SIBO and constipation. The most important question to be answered is whether it would be cost-effective to perform methane breath tests on all children with “functional” constipation, considering the relatively high prevalence of the disease. What we know from small scale studies is that children with constipation and retentive faecal incontinence are more likely to be methane producers than children with constipation alone (15, 20). Thus, possibly, these children may benefit from antibiotic therapy. However, large scale, prospective studies are warranted in order to (1) clarify the relation between methanogenic SIBO and constipation, and (2) determine whether children with methanogenic SIBO and constipation may benefit from antibiotic treatment.

### SIBO and Systemic Disorders

Increasing evidence suggests that SIBO may be implicated in the complex pathophysiology of stunted growth (6, 25) and environmental enteric dysfunction (EED; formerly known as environmental enteropathy) (24, 55, 56, 60). Stunting affects >30% of children under five from low income countries (118). As shown in Figure 3, intraluminal competition for micro and macronutrients between the excessive bacterial biomass and the host (119), as well as other SIBO-induced factors (e.g., diarrhoea, carbohydrate malabsorption, protein loss, increased intestinal permeability, intestinal and systemic inflammation), can lead to a negative caloric balance in the host, thereby, resulting in stunted growth and malnutrition. Such factors, too, characterise EED, thus it appears that SIBO may play an important role in EED's pathogenesis (6, 120). Vonaesch et al. (6) diagnosed SIBO in 96% of their patient cohort of children with stunting by the GHBT. In addition, Donowitz et al. (25) found a 16.7% SIBO prevalence in Bangladeshi children using the same diagnostic methodology. Taken together, these findings suggest a possible role of SIBO in stunting and EED's pathogenesis. Thus, it may be worthwhile to perform a hydrogen/methane breath test as part of the clinical approach to children with these diseases.

Moreover, although deficiencies of liposoluble vitamins (A, D, and E) and vitamin B 12 haven been documented in the adult population (121, 122), no studies have explored this issue in children with SIBO—a study assessing vitamin B12 and liposoluble vitamin status in children affected by SIBO would be of great interest. Menaquinone (vitamin K2) is produced by the gut microbiota (123), and thus from a physiological standpoint it would be contradictory to assume that vitamin K deficiency would arise in a bacterial-abundant environment. However, a recent case report described a 17-year-old female with vitamin K deficiency possibly caused by SIBO. The authors speculated that the vitamin K deficiency seen in this patient may have been the result of reduced menaquinone-producing bacteria, expansion of vitamin K-consuming bacteria, or severe malabsorption (124). Further gut metabolome studies (125) are needed to elucidate SIBO's role in vitamin deficiencies.

Currently, there are no guidelines regarding diagnosis or treatment of SIBO in children. Given the heterogenous and non-specific nature of the disease, it is sometimes challenging to decide in whom to initiate SIBO diagnostic work-up. However, it is of paramount importance to first rule out signs and symptoms (i.e., red flags) that may indicate diseases other than SIBO.

## Diagnosis

SIBO can be diagnosed by invasive and non-invasive methods. The non-invasive methods include breath tests, while invasive methods comprise culture-dependant and culture-independent approaches.

### Hydrogen and Methane Breath Testing

**Figure 4:**
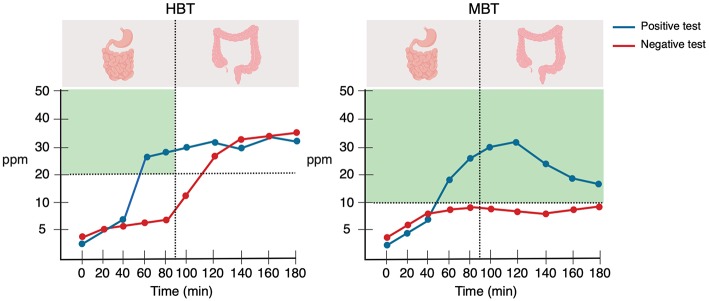
Graphical representation of the hydrogen and methane breath tests. The vertical dotted line indicates the completion of the orocecal transit time, and the horizontal dotted line indicates the current diagnostic thresholds for SIBO. The green shaded areas indicate where the test is considered positive. Created with BioRender.com.

Although subject to debate and controversy (126–128), the H_2_ and CH_4_ breath tests are increasingly being used due to their widely availability in healthcare facilities, because they are inexpensive, practical, and non-invasive (which is extremely important in paediatrics), and because the results can be interpreted on the same day of the test [the H_2_/CH_4_ breath test procedure is thoroughly reviewed elsewhere (92, 129, 130)]. According to the 2017 “Hydrogen and Methane-Based Breath Testing in Gastrointestinal Disorders: The North American Consensus”(92), a panel of 17 experts from North America and the latest consensus in regard to this topic, SIBO diagnosis is *suggested* when there is an increase in H_2_ of ≥20 ppm over baseline within the first 90 min of the test with either lactulose or glucose, or when there is an increase in CH_4_ of ≥10 ppm at any time point of the test (Figure 4). On the other hand, the older Rome Consensus (130) recommends the use of glucose as substrate due to its greater accuracy, and defines SIBO as an increase in H_2_ of ≥12 above baseline by using the GHBT. A definition of SIBO by using the LHBT was not included in this paper.

In order to understand the mechanisms behind H_2_ and CH_4_ breath testing, one must be aware of two concepts: orocaecal transit time and intraluminal gas production. The human body has no means of producing H_2_ and CH_4_ other than intraluminal microbial fermentation and methanogenesis, respectively (92, 130–132). As previously mentioned, the colonic microbiota represents virtually the whole gut microbiota, most of which is composed of fibre-fermenter anaerobes, mainly those belonging to the Actinobacteria and Firmicutes phyla (133). Undigestible carbohydrates reaching the colon are readily fermented by hydrogenogens (H_2_-producing bacteria) (134), and hence a physiological increase in H_2_ is expected in the colon. Intraluminal H_2_, in turn, is absorbed into the systemic circulation and is transported to the lungs where it can then be released through exhaled breath (129). Moreover, the orocaecal transit time—the elapsed time between ingestion of a substance until it reaches the caecum—has been shown to be around 90 min in both adults and children, as assessed by the LHBT (135). Thus, in the presence of SIBO, substrate administration will result in a small bowel H_2_ peak, occurring before the orocaecal transit time has completed (i.e., 90 min).

Glucose and lactulose are the substrates usually used for breath tests to diagnose SIBO. The former is a monosaccharide which is readily absorbed in the proximal small intestine, and the latter is a synthetic, undigestible disaccharide that reaches the caecum intact. In the presence of SIBO, glucose administration will result in a H_2_ peak which is produced in the small intestine (i.e., <90 min); in addition to this peak, lactulose administration can give rise to a second –colonic– H_2_ peak: the early peak produced by the bacterial overgrowth in the small intestine and the second one caused by the colonic microbiota fermentation (92, 129). However, both substrates have their own advantages and limitations. By using glucose, false negative results can arise in the presence of bacterial overgrowth in the distal small bowel (i.e., ileum), as glucose is readily absorbed proximally. In addition, an increased orocecal transit time can give rise to false positive results as the substrate reaches the colon rapidly, thereby undergoing premature fermentation by colon bacteria—this can occur with both glucose and lactulose (126). False negative results can also arise in the absence of H_2_-producing bacteria or in the presence of CH4-producing microorganisms (discussed below) (130). Based on a 2008 systematic review (136), the sensitivity and specificity of the GHBT ranged from 20 to 93% and 30 to 86%, respectively; and the sensitivity and specificity of the LHBT ranged from 31 to 68% and 44 to 100%, respectively.

*Methanobrevibacter smithii*, a member of the domain Archaea and the most abundant methanogen in the human alimentary tract produces CH_4_ as an end product of hydrogen metabolism (hydrogenotrophic methanogenesis), by using one molecule of carbon dioxide and four molecules of H_2_. The archaeon is extremely oxygen sensitive and relies completely on hydrogenogens for CH_4_ production, due to its inability to metabolise monosaccharides. For this reason, *Methanobrevibacter smithii* is considered an “obligate cross-feeder” (131). Furthermore, since the archaeon utilizes H_2_ to produce CH4, it can lead to a falsely negative HBT. Thus, it is recommended that both H_2_ and CH4 are measured in order to avoid this (92).

In conclusion, despite the limitations of the H_2_/CH_4_ test (128), it remains a useful tool in the diagnosis of paediatric SIBO due to its practical and non-invasive nature. Importantly, there are no reported significant side effects for the hydrogen/methane breath test other than transient abdominal pain or vomiting during the test.

### Culture-Dependant Approaches

Culture-dependent approaches are considered the current gold standard for definitive SIBO diagnosis. A small intestinal (i.e., duodenum or jejunum) aspirate is obtained at upper gastrointestinal endoscopy through a sterile catheter and stored anaerobically prior to culture for both aerobic and anaerobic bacteria. When using the proximal jejunal aspirate culture, a bacterial concentration of >10^3^ CFU/mL is regarded as indicative of SIBO (92, 109), although there is some heterogeneity in the literature in this regard and some experts recommend a threshold of >10^5^ CFU/mL as more specific (137). Despite being regarded as the gold standard diagnostic method, there are several notable limitations to this approach. Firstly, endoscopy-guided aspiration procedures are invasive, expensive, and require specialist input, limiting its availability and application in certain settings. Secondly, we know from microbiome research that standard clinical laboratory culture-based approaches have the potential to detect only a minority of the extant bacterial consortium. In addition, care must also be taken not to contaminate samples or sampling apparatus with oropharyngeal microbes, thereby contributing to false positives. Likewise, the significant heterogeneity in aspirate sampling protocols are known to impact substantially on the test outcome and accuracy. For example, one study previously used air, rather than nitrogen or carbon dioxide, during endoscopy to recover aspirate samples in suspected SIBO and cultured anaerobic bacteria from just one of 50 samples (138). Other variables include specific site of sampling and volume of aspirate. The lack of standardised protocol and diagnostic consensus for the gold standard investigation undoubtedly contributes to the massive variation in the SIBO prevalence and incidence rates reported in the literature. This, in turn, limits comparability between studies and ultimately hinders research progression in the field. In order for the development of a greater understanding and novel therapies for the disease to arise, the disparities in protocols and diagnostic criteria should first be addressed at a global scale. Finally, in the context of paediatric SIBO, endoscopy of seemingly healthy control children appears to present an obstacle in clinical research studies due to ethical considerations and, therefore, development of less invasive diagnostic approaches is warranted.

### SIBO in the Era of Next Generation Sequencing

As eluded to previously, culture-independent approaches based on NGS technologies have become the most widely applied modalities for studying both the composition and metabolic activity of the gut microbiota. There are several considerations to be addressed when selecting a methodology, including sample type, DNA extraction method, sequencing modality and platform, as well as bioinformatic pipeline [reviewed comprehensively in (43)].

In general, the composition of the gut microbiota is currently most cost effectively studied through 16S rRNA sequencing. The 16S region is a highly conserved region of rRNA within all taxa of bacteria, which displays sufficient variation and divergence in parts to allow differentiation at the genus level. Although it has been central to the progression of the field of microbiome research, 16S rRNA sequencing is crucially limited in the fact that it does not generate absolute data on quantities of bacteria, but rather provides investigators with a relative abundance of each taxa within a sample. The second modality which is indeed worthy of consideration is metagenomic shotgun sequencing. While 16S rRNA sequencing provides an overview of microbiota composition, metagenomic shotgun sequencing goes a step further by telling us who is there and what are they capable of in metabolic terms.

Although such sequencing capabilities are now commonplace in microbiological and medical research laboratories the globe over, uptake and application to SIBO research has been comparatively slow and, as a result, there is a dearth of relevant clinical data available. Despite this, several clinical studies which targeted alternate, but related, gastrointestinal disorders have provided some intriguing data on the disorder. In line with this, some small studies have reported on the gut microbiome of IBS cohorts, many of which were confirmed to suffer from concomitant SIBO. One such study investigated the faecal microbiota of a cohort of 30 Chinese patients with diarrhoea-predominant IBS, 14 of which were also confirmed to have SIBO by LHBT (139). At baseline, this cohort displayed reduced microbiota diversity, increased relative abundances of *Bacteroidetes* and decreased relative abundances of *Firmicutes* when compared to the healthy controls. In addition, the IBS cohort was shown to have reduced relative abundances of the genus *Lactobacillus*, as well as several genera associated with butyrate production. The investigators subsequently implemented a 2-week course of rifaximin, after which clinical symptoms improved and repeat LHBT demonstrated remission in 65% of the SIBO-positive subjects. However, the microbiota post-treatment displayed only minor alterations in taxa for which little metabolic information is available, a result which was mirrored previously in similarly designed trials of rifaximin in IBS (140, 141). Indeed, it must be reiterated that less than half of the IBD cohort studied in this trial were confirmed to have concomitant SIBO and the samples investigated (i.e., faeces) bears little resemblance to the site most relevant to the disease; therefore, no definitive conclusions on SIBO pathogenesis should be drawn from this data.

With regards to SIBO in children, one study outlined above aimed to investigate the microbiota in sub-Saharan children with stunted growth (6). In addition to faecal microbiota analysis, it was deemed pertinent to retrieve and analyse the microbiota of small intestinal aspirates, due to its role in nutrient absorption and malnutrition. The authors declared a form of microbial “decompartmentalization,” in which oropharyngeal-associated microbes were found to be over-represented in the small intestine of these children, 91% of which tested positive for SIBO. Although this was deduced primarily from culture-based methods, it is consistent with preliminary reports suggesting that SIBO is not caused by migratory colonic microbes (142). In turn, 16S rRNA sequencing of the duodenal microbiota in these subjects revealed a community containing near-equal parts *Proteobacteria* (32.4%), *Bacteroidetes* (29.6%), and *Firmicutes* (25.6%), with lesser portions of *Fusobacteria* (9.2%), and *Actinobacteria* (1.7%). Critically, however, this study was severely limited by the fact that there are no appropriate controls available for the microbiota analysis of duodenal aspirates, presumably due to ethical considerations. Therefore, we are left, once again, with half-truths and a degree of speculation on the potential role and composition of the small intestinal microbiota in SIBO.

These studies demonstrate that SIBO has been regarded largely as a sign or sequela of a gastrointestinal disorder, rather than a discrete disorder in itself. This perspective has meant that the pathogenesis of SIBO has sparsely been addressed directly, but rather has been of peripheral interest in studies with an intersecting interest. Having said this, perhaps the most informative characterisation effort of SIBO to date has come from a recent investigation of 126 adults displaying gastrointestinal symptoms (66 SIBO positive and 60 SIBO negative) (143). The authors uncovered that, although duodenal aspirate culture results do not correlate with symptoms, the aspirate microbiome was significantly altered in symptomatic participants. This altered microbiome is characterised primarily by decreased levels of the genus *Prevotella* and enhanced microbial metabolism of ascorbic acid. In addition, it was identified that age, recent antibiotic exposure, PPI use and diet were the major proponents in the disruption of the microbiome and onset of symptoms. In line with this, the investigators demonstrated that a dietary fibre restriction intervention in healthy high-fibre consuming individuals had significant effects on the microbiome and triggered gastrointestinal symptoms common to SIBO. This study identifies several potentially targetable components of SIBO pathogenesis and represents an excellent blueprint for the future study of the disease.

## Treatment

Due to the relative inaccessibility of duodenal samples for culture and difficulty in differentiating the culpable microorganism in a diverse ecosystem such as the small intestine, antibiotic therapy is generally initiated on an empiric basis. A 2013 systematic review and meta-analysis of antibiotic use in the context of SIBO found that rifaximin was by far the most commonly applied (144). While the meta-analysis ruled in favour of antibiotic use over placebo (effectiveness ratio 2.55, CI 1.29–5.04), rifaximin failed to reach a significant superiority (effectiveness ratio 1.97, CI 0.93–4.17). However, just three studies were deemed appropriate for this analysis and their heterogeneity limit the usefulness of this result. In line with this, the systematic review went on to reveal that monotherapy with 1,200 mg/d rifaximin was efficacious, at 60.8% remission. Moreover, the antibiotic reached a substantially heightened efficacy of 85% when combined with partially hydrolysed guar gum, albeit in a single trial. Two studies included in the systematic review investigated the use of metronidazole, demonstrating a return to normal breath test in 51% of SIBO patients; while a single small study reported remission in all 14 patients recruited and treated with ciprofloxacin.

A more recent systematic review and meta-analysis of rifaximin therapy in SIBO included data from >1,300 patients (145). A dose-dependent response was demonstrated for eradication rates and, in line with the previous systematic review, the most commonly used dose was 1,200 mg/d, with one study reporting 600 mg/d and another 1,600 mg/d. The investigators found an overall eradication rate of 70%, with adverse events reported in < 5%. In addition, in a subset of studies which assessed symptom severity and resolution, meta-analysis revealed remission from symptomology in 68% of patients who were found to be successfully eradicated of SIBO. These studies demonstrate the efficacy and safety of rifaximin in the treatment of SIBO and its symptoms.

While the studies included in the above meta-analysis were all conducted with adult cohorts, one study previously investigated the use of rifaximin in children (48). Applying a regimen of 600 mg/d for 1 week, the investigators reported a rate of 64% (*n* = 21/33) in breath test normalisation response. In light of the limited available data, a dose response study of rifaximin in a paediatric population would indeed be of interest. Finally, one study investigated the efficacy of a combination regimen of trimethoprim-sulfamethoxazole (TMP-SMT; 30 mg kg^1^ d^1^) and metronidazole (MTZ; 20 mg kg^1^ d^1^) twice daily for 2 weeks in slum-dwelling children suffering from SIBO (146). When retested for SIBO by breath test 1 month after commencement of this therapy, the authors noted 95% (*n* = 19/20) resolution of the disorder. However, the lack of a placebo or non-intervention control group limits assessment of temporal effects on SIBO status. Taken together, these results indicate that antibiotics are an effective means of treating SIBO in children and, although non-rifaximin antibiotics appear to produce greater resolution, the data is weaker for such medications and rifaximin has demonstrated safety in a greater number of participants and studies.

While antibiotics remain the first-line and gold-standard approach to SIBO management, there are additional or alternative approaches that may have application in the future, but the efficacy of which remains uncertain at present. For instance, there is biologic plausibility to the hypothesis that there may be a role for low FODMAP (Fermentable Oligosaccharides, Disaccharides, Monosaccharides, and Polyols) diets in decreasing fermentable substrates in the context of SIBO. Low FODMAP diets aim to greatly deplete or entirely eliminate the highly fermentable simple carbohydrates which are commonly found in certain dairy products, fruits, vegetables, nuts and legumes, with the ultimate aim of graded reintroduction of specific FODMAPs to elucidate the culpable source (147).

Interestingly, probiotics have also previously been considered as potential agents in the management of SIBO (148). In line with this, a more recent meta-analysis evaluating the efficacy of this avenue revealed that probiotic intervention resulted in vastly reduced H_2_ levels (WMD −36.35, CI −44.23−28.47) and increased rates of decontamination (RR 3.29, CI 1.47–7.36) when compared to placebo. More intriguingly, however, is that probiotics also demonstrated more favourable results when compared directly to metronidazole treatment (RR 1.49, CI 1.07–2.08). Despite this, the best results overall were obtained when probiotics were combined with rifaximin or minocycline.

Finally, there is some evidence to suggest that certain statins may have a role in depleting *Methanobrevibacter* spp., thereby offering a novel therapy for methane-specific SIBO (149, 150). In line with this, a modified-release formulation of lovastatin, termed SYN-010, has been created to deliver the drug in a biphasic manner during transit, thereby avoiding considerable degradation and absorption in the upper gastrointestinal tract (151). A dose of 42 mg/day of SYN-010 for as little as seven days was demonstrated to significantly reduce methane production when compared to placebo in a multi-centred double-blind RCT (152). Moreover, this promising therapy recently entered an efficacy and safety trial in patients with IBS (ClinicalTrials.gov ID: NCT03763175). The effect of this drug appears to be primarily due to the HMG-CoA reductase inhibition attributes of statins, thereby preventing the formation of mevalonate, a primary precursor in of key membrane lipids specific to archaea. This mechanism, along with several others, has been previously reviewed extensively elsewhere (41), and so, will not be discussed further herein.

## Prognosis

At present, it is unclear whether the gut microbiota changes that characterise paediatric SIBO have detrimental effects in the long-term. In order to shed light on the long, and even short-term prognosis of children with SIBO, large scale, prospective studies are warranted.

## Conclusion

Herein, we attempted to synthesise the most current literature on paediatric SIBO, from a gut microbiota perspective. Paediatric SIBO is a heterogenous and poorly understood entity, whose prevalence and incidence are difficult to determine due to the lack of uniformity and consensus of its diagnostic criteria. However, based upon the available literature, it appears that SIBO is a common underlying diagnosis in children who present clinically with certain FGDs and stunted growth, and in children with history of acid suppressive therapies, intestinal motility disturbances, anatomical alterations, or impoverished conditions. Further research with integration of culture-dependant and culture-independent approaches is needed in order to (1) understand paediatric SIBO pathogenesis, clinical presentation, and prognosis, and (2) establish global consensus on the diagnostic criteria for SIBO.

## Author Contributions

DA contributed conception and design of the manuscript. DA and PR wrote the first draft of the manuscript. ET, JR, and EQ wrote and edited sections of the manuscript. All the authors contributed to manuscript revision, read, and approved the submitted version.

### Conflict of Interest Statement

The authors declare that the research was conducted in the absence of any commercial or financial relationships that could be construed as a potential conflict of interest.
